# Health care in future community: innovatively discover and respond to the needs of today’s seniors

**DOI:** 10.3389/fpubh.2023.1302493

**Published:** 2023-12-12

**Authors:** Bowen Zhou, Qidan Deng, Shiyuan Zhou, Dongni Zhuo

**Affiliations:** ^1^School of International Economics and Trade, Jilin University of Finance and Economics, Changchun, China; ^2^School of International Economics and Trade, Jilin University of Finance and Economics, Changchun, China; ^3^Department of Computer Science, University of Toronto, Toronto, ON, Canada; ^4^College of Education, University of Washington, Seattle, WA, United States

**Keywords:** health care, future community, aging population, digital economy, information technology

## Abstract

**Introduction:**

In the context of the digital economy, the emergence and application of emerging technologies have accelerated the integration of traditional social structures with new technologies, leading to the inception of the “Future Community” as an innovative urban unit. With an aging population’s rapid and sustained rise, integrating health care for older adults with modern information technology is gradually moving towards holistic governance. This approach utilizes the Future Community as a medium and aims for quality enhancement and increased efficiency, which instrumentally addresses the diversified health care needs of China’s aging era.

**Methods:**

In this study, we employed a questionnaire survey method that covered 11 communities in Tianjin City to understand better the current status and characteristics of their health care services.

**Results:**

The survey results show that the means of community health care for older adults are gradually being upgraded, and the demands are shifting. Then, we arrive at three conclusions: firstly, technological innovation and smart approaches have the potential to positively influence the quality of health care in these communities. Secondly, allocating health care resources within communities can have a salutary effect on the psychological well-being of seniors. Thirdly, actively involving seniors in community life and governance can elevate their self-worth.

**Discussion:**

At last, in conjunction with current challenges, we think that deepening multi-party collaboration, educating specialized talents, and bridging the “digital gap” would be effective ways to establish a future community for seniors.

## 1 Introduction

The aging of the population and informatization are two significant trends in contemporary societal development. Evidence from the seventh national census indicates that China’s elder population aged 60 and above has reached 264.02 million, accounting for 18.7% of the total population, an increase of 5.38 percentage points since 2010 (1). During the “14th Five-Year Plan” period, people born during China’s first and second baby boom phases will join middle and older age groups, precipitating a substantial increase in care needs (2). Accompanying the rapid growth of an aging population, there’s a pervasive societal concern over the scarcity of health care resources. Concurrently, the demand structure among older adults is shifting from subsistence-based to development-based: health care shall not only deliver services but also offer comprehensive, multi-scenario solutions in line with the aging process (3). The emerging demand for modern health care services challenges the unbalanced and insufficient development of traditional care services (4).

In recent years, China has placed significant emphasis on the development of the digital economy. The “Outline of the 14th Five-Year Plan for National Economic and Social Development and the Long-Range Objectives Through the Year 2035” stresses leveraging digital technologies to foster new advantages in the digital economy and to facilitate the integration of fields, including economy, livelihood improvement, and social security. The successful integration of the digital economy into daily life requires both vehicle and demand, leading to the emergence of innovative urban units in the digital economy—the “Future Community.” The Future Community is the vehicle of digital economic development, a platform for integrating societal resources and extending industry chains, and epitomizes the “last mile” of community governance (5). It fosters collaboration among governments, enterprises, communities, and residents, establishing a full-cycle service chain to ensure precise service provision and comprehensive oversight. Big data facilitates optimized allocation of different societal resources, making services support the move towards “integrated governance” by connecting and sharing data (6). The infusion of “intelligence” is the foundational philosophy behind the construction of the Future Community throughout health care for older adults. Digital management should be combined with multiple elements, such as spatial considerations, industry practices, and institutional systems (7). This facilitates the provision of real-time, convenient, efficient, cost-effective, and intelligent health care for older adults service. By adeptly addressing the diverse and multifaceted needs of the older adults, it aims to optimally address the challenges associated with health care and support.

The smart communities of the United States are the earliest exploration of the “Future Community” concept. The U.S. integrated advanced technologies into health care by establishing a health care for older adults service system encompassing “Big data analysis—Remote medical service systems—Artificial intelligence caregiving (8, 9).” Singapore’s “Smart Nation” initiative advocates that health care for older adults service should utilize smart technologies while incorporating humanistic care, aiming to establish a proactive and healthy aging society (10, 11). Japan, grappling with severe aging challenges, places significant attention on the establishment of cross-disciplinary smart communities in telecommunications, healthcare, education, and wellness, utilizing informatization technologies to provide comprehensive and intensive care for the older adults (12). Under the concept of smart communities, China introduced the idea of the “Future Community.,” making human-centricity, ecological harmony, and digitalization crucial concepts. Through technological empowerment, space creation, and institutional innovation, China seeks to optimize community health care for older adults service, thereby constructing a holistic wellness-oriented future health environment (13).

The progression of the digital economy is concomitant with the advent of a moderately aging society. Digital technologies are boosting the transformation and advancements in the health care service domain. Utilizing the Future Community as a platform and aiming for enhancement in quality and efficiency, the fusion of health care for older adults services with information technology is an essential sector of resource reallocation in the trajectory of digital-era development. It represents a crucial approach for China’s aging society to reflect on the diverse health care needs of the aging era. The imperative questions include how to construct a community platform in the digital economy optimally, respond to the different levels of needs of the current large elder population, and provide multi-level health and aging services. Research should be deepened into health care for older adults within the context of the Future Community.

## 2 Literature review

In the fields of smart healthcare for the older adults and future community development, research has garnered increasing attention within the academic community. Studies in this domain underscore the application of smart technologies, including data analytics and artificial intelligence, to deliver more specialized health care services (14). The direction of future community development includes the integration of information resources within regions, enabling the digitization of health scenarios to enhance the intelligence of health care services. Simultaneously, this involves the establishment of a 5G medical ecosystem, facilitating resource-sharing between high-quality hospitals and community clinics (15). Furthermore, the concept of digital technology empowerment has gained widespread recognition, combining online technologies with offline governance to create a streamlined digital management platform ensuring the safety of older adults in the community (16). From the perspective of spatial production theory, research also explores the production of physical spaces in future communities, necessitating extensive technological research and development, including the support of the Internet of Things and big data for the establishment of a city-wide intelligent system. This system closely monitors the dynamics of elder individuals through smart devices, ensuring greater convenience in their daily lives (17).

In the field of community-based eldercare, the application of digital technology has emerged as a pivotal factor. This encompasses leveraging digital technology to expand service offerings, facilitate social interaction, and provide online psychological support to positively impact the mental well-being of seniors, thereby enhancing their overall life satisfaction and quality (18). Simultaneously, the design of entertainment and educational facilities within future communities is poised to play a crucial role. The fusion of “entertainment + technology” and “education + technology” not only fosters deeper engagement and cognitive experiences among community residents with public amenities but also offers convenience for the leisure activities and re-education of older adults, catering to their spiritual and cultural needs, ultimately elevating the community’s overall happiness index (19). Moreover, the application of scene theory in the development of future communities involves the creation of diverse scenarios by combining various comfortable facilities to meet the multifaceted needs of senior citizens, enriching their spiritual and cultural lives (20).

This emerging trend underscores future communities’ ecological and intelligent development, aiming to create a novel “production–life–ecology” coupling model (21). This model encourages the active participation of senior citizens throughout the entire process of community planning, construction, and operational management. Simultaneously, with a focus on the guiding principles of “public participation” and “digital empowerment,” communities are poised to fully leverage their home-ground advantage. They are expected to empower the elder population with digital capabilities, enhance their proficiency in utilizing digital tools, and further enrich their social lives. This approach encourages seniors to participate more actively in community life and governance, fostering a comprehensive integration of “self-governance” and “service orientation.” It not only caters to individual self-realization but also nurtures a sense of community, belonging, and identity (22, 23).

In summary, the current academic research focus centers on smart healthcare for the older adults and the development of future communities. Numerous scholars have presented their respective viewpoints and approaches. Scholars believe that technological innovation, healthcare resource allocation, and the active involvement of the older adults in community life and governance are of significant importance for the older adults’ health care levels, psychological well-being, and self-worth enhancement. Building upon the aforementioned research, the following questions are posed: Will technological innovation and intelligent means in future communities positively impact the health care levels of seniors? Can the allocation of healthcare resources in the community effectively enhance the psychological well-being of seniors? Does the active engagement of seniors in community life and governance promote their self-worth?

## 3 Materials and methods

### 3.1 Questionnaire participants

In Tianjin City, a survey was conducted targeting 502 residents aged 50 and above from 11 different communities, namely: Heshun Dongyuan, Heshun Xiyuan, Junli Garden, Junhua Garden, Junxiu Garden, Junxiang Garden, Junrui Garden, Dongmei Xuan, Xiahe Xuan, Chunzhu Xuan, and Zhao Guli. Prior to the survey, the inclusion criteria and research objectives were explained to all volunteers. Eligible individuals were those residing in these communities who met the age criterion of 50 years or older, possessed a clear understanding of the research objectives, and were capable of effective communication.

In this study, we set the age criterion at 50 years and older, which differs from the traditional definition of old age by the World Health Organization, set at 65 years. However, this choice was made considering the local demographics, while also taking into account individuals who have retired early or are about to retire. In fact, early retirement has become a common trend in many regions, and this group of individuals also faces various challenges associated with aging. Therefore, understanding their needs is crucial for a comprehensive understanding of the characteristics and requirements of the elder population. Choosing this age criterion has a certain rationality in the research design, aiming to ensure coverage of different older adults groups, enabling a more comprehensive exploration of the health care needs of seniors.

It is noteworthy that 600 questionnaires were distributed during the on-site survey, and all 600 were returned. However, given that the survey targeted senior residents, it is important to acknowledge that filling out questionnaires may have presented challenges for some participants due to potential difficulties associated with age. As a result, during the subsequent data analysis, it became evident that some of the responses contained logical errors or were incomplete. Therefore, data from 502 residents were ultimately included in the analysis, leading to a questionnaire effective rate of 83.67%.

### 3.2 Questionnaire design

The survey questionnaire consisted of two sections. The first section asked respondents’ demographic information, capturing basic data on the respondents, such as age, gender, living situation, and monthly household income. The second section delved into the respondents’ experiences and needs towards community-based health care services. It primarily assessed how much community health care services they experienced and their degree of need for such services. Through this questionnaire-based investigation, the goal was to identify which specific needs of the elder population could serve as motivations for the development of community health care. Additionally, it aimed to reveal the most appropriate strategies for the community to address and meet these needs.

Assessing the seniors’ perspective on technological innovation and smart solutions in community-based health care based on questions like “I believe that it is necessary for the community to provide healthcare for the older adults and infrastructure.” “I am willing to provide electronic health records and data to the community or relevant health care institutions.” “I believe that smart facilities can bring more convenient and efficient healthcare management and nursing services.” and “I believe that community-provided smart information services and smart on-demand services can enhance personal health care.”

Evaluating the senoirs’ satisfaction with various types of health care services based on questions such as “I believe that harmonious neighborly relationships help reduce my sense of loneliness and increase social participation.” “I think recreational and educational health care services help broaden personal interests and enrich life experiences.” “I believe that educational services can provide opportunities for knowledge sharing and communication, enriching my spiritual life” and others.

Determining the aspects that should be prioritized when providing health care services in the community based on the question “Which aspects of health care services should the community consider first?”

Understanding the seniors’ expectations for improvements in various aspects of health care services and facilities in the community based on questions such as “Which of the following four types of health care services do you believe the community should increase in terms of frequency or quality?” and “If the following public facilities in the community were adapted for the older adults, what is your level of demand?”

### 3.3 Descriptive statistics

Table 1 presents the basic demographics of the survey respondents of the individuals who participated in this questionnaire: 265 were male (52.79%), and 237 were female (47.21%), indicating a relatively balanced gender distribution among respondents. The age distribution of the senior respondents was rather even, with 51% aged 50–60 years, 22.50% aged 61–70 years, 11.90% aged 71–80 years, and 14.54% over 80 years of age. The survey specifically targeted the elder population, as they are the primary beneficiaries of community health care services and are generally well-informed about the current services in the community. This demographic focus contributes positively to a deeper understanding of community Health care service provisions.

**Table 1:** Descriptive analysis of the basic information of the respondents.

Statistical content	Variables	Number	Proportion
Gender	Men	265	52.79%
	Women	237	47.21%
Age	50–60	256	51%
	61–70	113	22.50%
	71–80	60	11.90%
	> 80	73	14.54%
Education level	Primary school or lower	217	42.23%
	Middle school education	123	24.50%
	High school education/Secondary vocational school education/Industrial school education	102	20.32%
	Three-year college education	42	8.37%
	Bachelor’s degree	16	3.19%
	Postgraduate education or higher	2	0.40%
Employment status	Retirement	160	31.87%
	Employment	184	36.65%
	Unemployed	63	12.55%
	Re-employment	95	18.92%
Total monthly income	< 3,000	141	28.09%
	3,000–5,000	205	40.84%
	5,000–7,000	81	16.14%
	> 7,000	75	14.94%
Proportion of monthly pension expenses	< 10%	57	11.35%
	10–30%	185	36.85%
	30–50%	136	27.09%
	> 50%	124	24.70%
Living conditions	alone	51	10.16%
	With spouse	227	45.22%
	With children	70	13.94%
	With spouse and children	129	25.70%
	Domestic workers and others	25	4.89%
Health status	Physical capacity intact	346	68.92%
	Mild disability	77	15.34%
	Moderate disability	50	9.96%
	Severe disability	24	4.78%
	Completely disabled	5	1%

The educational background of the older individuals was also diverse: 42.23% had primary education or below, 24.50% had middle school education, 20.32% had high school education, secondary vocational school education, or industrial school education, 8.37% had a three-year college education, 3.19% had a bachelor’s degree, and only 0.40% had postgraduate education or higher. The majority of their educational levels are concentrated at middle school or below, secondary vocational school education, and industrial school education.

In terms of employment status, 31.87% of the older adults were retired, 36.65% were employed, 12.55% were unemployed, and 18.92% had retired but backed to work, which means 55.57% of the older adults maintained an employment status. Income distribution among the older adults was relatively even: 28.09% earned below 3,000 yuan, 40.84% earned between 3,000–5,000 yuan, 16.14% earned between 5,000–7,000 yuan, and 14.94% earned above 7,000 yuan, indicating a relatively high-income level.

The distribution of health care expenditure was comparatively balanced: 11.35% spent less than 10% of their income, 36.85% spent between 10 and 30%, 27.09% spent between 30 and 50%, and 24.70% spent over 50%. Living arrangements varied among elder individuals from different households: 45.22% lived with a spouse, 13.94% lived with their children, 25.70% lived with both a spouse and children, 10.16% lived alone, and 4.89% lived with a caregiver or others.

Regarding the physical health status of the older adults, 68.92% were fully able-bodied, 15.34% had mild disabilities, 9.96% had moderate disabilities, 4.78% had severe disabilities, and 1% were completely disabled. A significant proportion of the elder population demonstrated a good degree of self-sufficiency.

## 4 Results

### 4.1 The means of services are gradually upgrading

Examining the evolution of future community development, the initial stages of “smart” community-based health care were predominantly understood as hardware investments. The emphasis was primarily on upgrading hardware equipment, such as network infrastructure, monitoring equipment, and smart eldercare devices. However, this approach, when implemented in practice, can result in a low level of scene integration within the community. In other words, these intelligent hardware devices may not fully assimilate into the daily operational scenarios of the community and the genuine needs of the senior residents. Contemporary future communities now encompass both hardware facilities and software services. By centering on the concept of health intelligence and smart healthcare, these medical institutions employ digital thinking to dynamically manage the health conditions of community senior residents, progressively actualizing future community health environments. As demonstrated by the survey results (Table 2), 55.38 and 43.43% of the older adults expressed “very satisfied” and “quite satisfied” responses, respectively, to smart facilities in the community. Seniors utilize health monitoring devices to keep track of their health status and employ one-touch emergency devices to access urgent rescue services (24). Communities have integrated monitoring data of older adults’ smart devices, like smartwatches, with digital family doctors and established collaborations across health and social services, realizing a unified digital medical care system. 61.75 and 37.05% of the older adults conveyed “very satisfied” and “quite satisfied” sentiments towards the intelligent platforms constructed within communities. They can glean health knowledge from social platforms, place meal orders, book home services, and schedule medication deliveries via the community online platform. Communities have achieved multifaceted Apps that bridge medical insurance, the internet, local stores and hospitals, and more, introducing remote consultations, mobile hospital referrals, and drug deliveries, achieving full digitalization of in-home healthcare.

**Table 2:** Satisfaction with technological innovation and intelligent means of community health care.

Projects	Option	Number	Proportion	Cumulative proportion
Intelligent facilities	very satisfied	278	55.38%	55.38%
	quite satisfied	218	43.43%	98.81%
	uncertain	6	1.20%	100.00%
	unsatisfied	0	0.00%	100.00%
	very unsatisfied	0	0.00%	100.00%
Intelligent platform	very satisfied	310	61.75%	61.75%
	quite satisfied	186	37.05%	98.80%
	uncertain	6	1.20%	100.00%
	unsatisfied	0	0.00%	100.00%
	very unsatisfied	0	0.00%	100.00%

Historically, eldercare services were mainly characterized by direct human interaction or basic human-computer interactions. Present and future health care services are gradually leveraging cloud computing centers, cutting-edge multi-party interaction, and backend data support, ensuring precision, professionalism, human-centeredness, and intelligence in multi-level eldercare. Concurrently, under the aegis of artificial intelligence, there is an in-depth understanding of human perception and cognitive states, constructing an intelligent human-computer interaction framework with “human in the loop.” Through advanced sensing technologies like facial or voice recognition, comprehensive analysis is conducted on seniors’ physical conditions, physiological and psychological changes, and social adaptability, among other factors. This helps ascertain the structural and dynamic trends of older adults’ needs, accurately gaging the subjective needs and attitudes of seniors who are adapting smart health care services. This breakthrough overcomes the emotionless character of smart devices and virtual systems, enabling computers to make more autonomous decisions, catering to the older adults’ needs (25). In health care services, interactive technologies will ultimately achieve a seamless integration of intelligent technology and human warmth (26).

### 4.2 The demands for the services are changing

Due to the heterogeneity within the elder population, individuals of the same age may have distinct physical conditions. While some seniors may have already lost the capacity for basic daily activities, necessitating comprehensive care, others may remain physically robust and mentally healthy, enabling them to continue actively contributing to society. This underscores the importance of regarding the older adults as a valuable resource rather than a societal burden, with a focus on acknowledging their intrinsic worth. In alignment with the strengths perspective theory, this theory focuses on harnessing an individual’s inherent strengths and resources. Within the elder population, the application of this theory ignites personal initiative, enabling them to proactively address potential challenges and adversities, ultimately attaining personal and societal goals.

**Figure 1:**
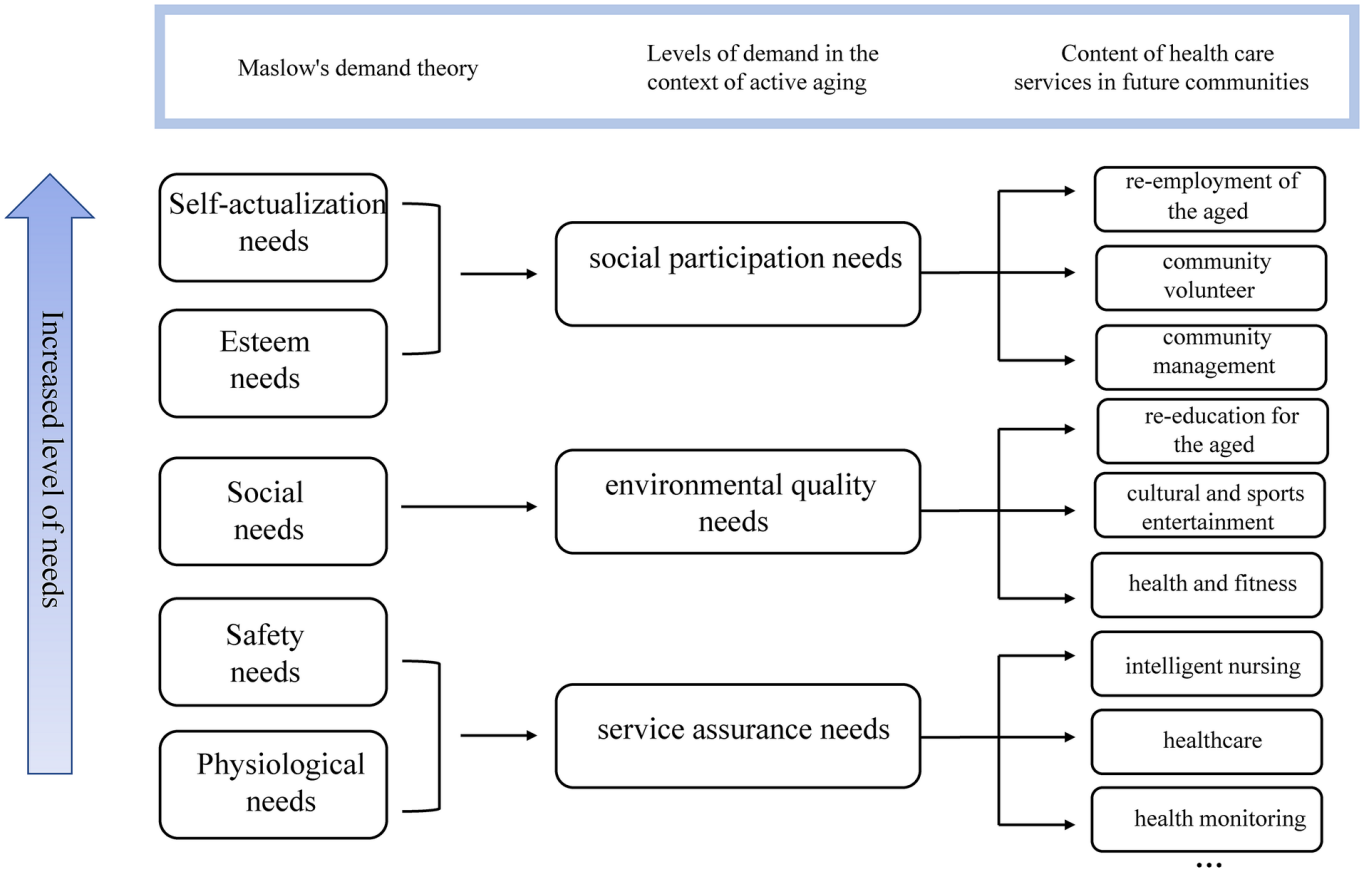
Content of the needs of older adults based on Maslow’s hierarchy of needs.

The construction of health care environments in future communities is centered around the comprehensive service needs throughout the community life cycle. By focusing on collecting the needs of the older adults, it inherently embodies human-centric values and compassionate care. Drawing upon Maslow’s Hierarchy of Needs, the layers of older adults’ needs in the context of active aging are categorized as service assurance needs, environmental quality needs, and social participation needs, as shown in Figure 1.

1. Service Assurance Needs refer to material needs such as health and daily life care, aiming to fulfill the physiological and safety needs of the older adults.
2. Environmental Quality Needs involve providing a comfortable living environment for the older adults, emphasizing the construction of community landscapes, entertainment, and other spatial environments to meet their social needs on a psychological level (27).
3. Social Participation Needs denote providing opportunities for the older adults to engage in societal activities, enhancing their sense of self-worth, community belonging, and societal integration. This is to fulfill their intrinsic needs for respect and self-actualization.

According to the survey results (Table 3), 98.80% of the older adults expressed satisfaction or higher regarding the community’s services meeting their Service Assurance Needs. Seniors can avail of refined wellness services through the community-established public health and nursing systems. The community has transformed the digital health platform into an “Internet + Health” intelligent entity, constructing a service system encompassing online appointments, A.I. doctor consultations, and home-visiting family doctors to meet the daily medical needs of the older adults. The community has constructed a comprehensive smart health care nursing service platform based on Internet and Artificial Intelligence (A.I.) technologies and has vertically established a novel smart nursing plan that encompasses the home, the community, and hospitals (19). The primary objective is to create a nursing facility system characterized by the “Internet + Nursing services with regional coordination.” Smart wearable devices worn by senior citizens will also be connected to digital health smart monitoring stations and nurse-on-call packages. Through this, community care centers can monitor elder individuals’ data anomalies and provide doorstep nursing services. This system enables seniors to request online and receive convenient and professional nursing services in their homes.

**Table 3:** Satisfaction with the supply of various types of aged care services in the community.

Option	Very satisfied	Quite satisfied	General	Unsatisfied	Very unsatisfied
Service assurance needs	294 (58.57%)	202 (40.24%)	6 (1.20%)	0 (0%)	0 (0%)
Social participation needs	310 (61.75%)	183 (36.45%)	9 (1.79%)	0 (0%)	0 (0%)
Environmental quality needs	239 (47.61%)	156 (31.08%)	26 (5.18%)	53 (10.56%)	28 (5.58%)

78.69% of the older adults had satisfaction or higher with the community’s provision of health care services that address their Environmental Quality Needs. The community empowers data for public sports facilities like pocket gardens, intelligent fitness equipment, smart sports venues, and elder-friendly parks to monitor and manage the older adults’ walking, fitness, and other training activities, thereby promoting the integrated development of older adults health and fitness. Community cultural centers, digital libraries, senior clubs, and card game rooms leverage cloud computing and mobile internet technologies to pioneer an innovative “culture + technology” model. This approach actualizes continuing education for seniors and offers leisure and entertainment activities, fulfilling their cultural and spiritual needs for a pleasant living environment.

98.21% of the older adults conveyed satisfaction or higher with the community’s provision of health care services that meet their Social Participation Needs. Communities are working towards creating and developing ways of employment for the older adults, offering opportunities for re-employment and volunteer services. Moreover, adhering to the thoughts of co-construction and co-sharing, management of community public affairs, such as community libraries, senior universities, and cultural centers, requires the involvement of diverse entities. As part of the community body, seniors also have opportunities to contribute to community management. By leveraging modern information technology to counteract the physical limitations of seniors, they can use their rich experiences to create value for the community, fostering a pleasant community ambiance.

To ascertain the priorities of communities in providing health care services, we asked respondents to rank the importance of various health care services. Based on the survey results (Table 4), we found that seniors have varying degrees of focus on different categories of community health care for older adults. Specifically, the overall score for Service Assurance Needs was 2.57, ranking first, followed by Social Participation Needs with a score of 1.74, ranking second, and Environmental Quality Needs with a score of 1.69, ranking third. These findings reflect that when considering community health care services, seniors primarily focus on meeting basic life requirements while also expressing higher-level needs such as environmental quality and social participation. Community-based health care for older adults continue to innovate and improve, becoming more comprehensive. These services are expected to fulfill not only the basic physiological needs of seniors but also their psychological needs and self-realization aspirations.

**Table 4:** Priority ranking of community health care services to meet various needs.

Option	comprehensive score	The first	The second	The third	Subtotal
Service assurance needs	2.57	330 (65.74%)	128 (25.5%)	44 (8.76%)	502
Social participation needs	1.74	68 (13.55%)	236 (47.01%)	198 (39.44%)	502
Environmental quality needs	1.69	104 (20.72%)	138 (27.49%)	260 (51.79%)	502

Survey results (Table 5) indicate that 34.06% of the elder population desire an increased provision of lifestyle-related services. Through community service offerings or enhanced hardware facilities, seniors can access services such as smart information, online appointment systems, and intelligent transportation services. This underscores a broader aspiration among the older adults to acquire more convenient and advanced lifestyles from community support, subsequently enhancing their overall quality of life. Specifically, the community adopts an intelligent information delivery mechanism in order to meet the needs of senior residents for notification of community activities, weather forecasts, news information, and social interaction. Those demands can be met through various technological means, including smart screens, mobile applications, and voice assistants, to help seniors rapidly access pertinent information. Additionally, seniors can easily schedule housekeeping, repair, and dining services through community-based smart applications to meet their daily living needs. These services predominantly depend on online platforms and digital channels, allowing seniors to efficiently procure on-demand services. Lastly, the community helps seniors make their daily trips more convenient by providing shared mobility tools, electric mobility scooters, smart navigation systems, and personalized trip-planning services.

**Table 5:** Expectations for improving the supply of community pension services.

Projects	Option	Subtotal	Proportion
In the following four categories of health care services, in which aspect do you think the community should increase the number of services or service quality	Lifestyle-related services	171	34.06%
	Medical service	169	33.67%
	Cultural and entertaining services	102	20.32%
	Mental health services	60	11.95%

33.67% of the older adults expressed a desire for improving medical services, signaling a greater demand for community-provided emergency rescue services, intelligent caregiving, and smart medical services. Initially, communities establish intelligent emergency rescue systems, utilizing smart emergency call buttons, intelligent monitoring equipment, and remote surveillance to ensure seniors receive timely assistance in urgent situations, thus protecting their lives. Furthermore, through the inception of digital medical platforms, communities provide seniors with remote health monitoring, medication management reminders, and self-designed health guidance, guaranteeing a holistic and effective self-health management approach. In conclusion, leveraging digital medical platforms and integrated smart health care platforms, communities offer seniors remote medical consultations, online diagnoses, and medical appointment services, ensuring rapid and convenient medical care. These medical care services in the community, through the application of digital technology, provide more comprehensive, timely, and convenient healthcare solutions for the older adults, helping to maintain their health, safety, and quality of life. The increase in demand for health care services for the older adults further highlights the fact that their need for health protection has expanded from simple medical care to more comprehensive health management.

20.32% of the older adults are keen on an increased offering of cultural and entertaining services, while 11.95% express an interest in mental health services, highlighting their emphasis on psychological needs and self-worth actualization. For instance, they seek spiritual entertainment, cultural experiences, and social participation. This result emphasizes the need for community-based healthy aging services that not only focus on physical health but also provide more opportunities and support to meet the spiritual and social needs of seniors.

The survey results, as detailed in Table 6, reveal that seniors have varying priorities regarding the age-friendly reconstruction of community public facilities. 93.03 and 92.83% of the respondents expressed a pronounced demand for the age-friendly renovation of community healthcare and nursing facilities, underscoring the pressing and paramount significance of refurbishing these establishments. The following aspects are sports facilities and cultural entertainment facilities, with 78.88 and 72.51% of the older adults signifying an urgent need for age-friendly modifications, respectively. However, the demand for the improvement of educational facilities was comparatively lower, with 43.03% of the seniors articulating a requirement in this domain. Given the scarcity of community resources, a phased renovation strategy can be adopted. The initial phase will prioritize the refurbishment of healthcare and nursing facilities, encompassing health service stations, community hospitals, senior service centers, and day-care centers, targeting advancements in their intelligent capabilities. This aims to satisfy the older adults’ fundamental physiological needs, safety requirements, and healthcare maintenance. The subsequent phase will shift its focus to the upgrade of sports and cultural entertainment facilities. This includes enhancements to smart fitness equipment, community intelligent sports venues, community parks, cultural entertainment hubs, and senior game rooms, all aimed at improving the quality of life and fostering social interaction for the older adults. For educational facilities, such as universities for the older adults, digital libraries, and wellness learning centers, decisions on their renovation can be contingent on specific circumstances, ensuring optimal utilization of community resources. Given the constraints on resources, this strategy is poised to address the needs of the older adults to the greatest extent while simultaneously improving the overall age-friendly standard of the community. As seniors’ expectations for community living quality continue to increase, communities must continually adapt to the evolving needs of the older adults, continuing innovations and enhancements, with the objective of providing more comprehensive, accessible, and intelligent health care services.

**Table 6:** The demand for aging transformation of various public facilities in the community.

Option	Very necessary	Comparison necessary	General	Unnecessary	Completely unnecessary
Healthcare facilities	467 (93.03%)	32 (6.37%)	3 (0.6%)	0 (0%)	0 (0%)
Nursing facilities	466 (92.83%)	31 (6.18%)	5 (1%)	0 (0%)	0 (0%)
Sports facilities	396 (78.88%)	67 (13.35%)	38 (7.57%)	1 (0.2%)	0 (0%)
Cultural and entertainment facilities	364 (72.51%)	95 (18.92%)	36 (7.17%)	7 (1.39%)	0 (0%)
Educational facilities	216 (43.03%)	79 (15.74%)	170 (33.86%)	29 (5.78%)	8 (1.59%)

## 5 Discussion

For additional requirements for specific article types and further information, please refer to “Article types” on every Frontiers journal page. In the future communities, health care encapsulate the aspirations of the seniors for a great elder life. Technological innovation and smart approaches have the potential to positively influence the quality of health care in these communities. This encompasses the deployment of cutting-edge technologies such as data analytics, artificial intelligence, and the Internet of Things, which can facilitate more specialized health care services, ensuring a more convenient and secure daily life for seniors. Allocating health care resources within communities can have a salutary effect on the psychological well-being of seniors. Enhancing age-appropriate design in community public facilities and enriching the cultural and spiritual life of seniors can bolster their psychological comfort and sense of happiness. Actively involving seniors in community life and governance can elevate their sense of self-worth. As an important social and management place, communities can provide seniors opportunities to participate in community events and decision-making processes, thereby reinforcing their sense of community belonging and self-esteem. The future of communities is, in essence, already upon us. In the digital economy era, there’s significant potential in vigorously advancing the construction of health care within future communities as the fundamental service unit for “Smart City” governance. Integrating health care strategies within these communities represents a paramount direction for contemporary development.

Certainly, when discussing the future of health care for older adults in communities, we advocate for the crucial cultural shift of “active aging.” This transformation encompasses several pivotal components. Firstly, from the perspective of the senior individual, it encourages proactive aging and underscores that the process of aging offers opportunities for continual personal growth and active participation in life. This paradigm shift nurtures a culture that values the contributions of seniors and recognizes their enduring potential for personal development. Additionally, this paradigm grants seniors autonomy, empowering them to make choices about their lifestyle and caregiving preferences, ensuring that they have a voice in decisions that affect their well-being and daily lives. It also emphasizes lifelong learning and personal development for the older adults, encouraging them to develop interests and hobbies, engage in community activities, and help them learn to use innovative technologies to bridge the digital gap.

Secondly, from a societal standpoint, active aging underscores the importance of fostering an age-friendly environment within communities, reflecting a culture that respects and encompasses the well-being of senior community members. Furthermore, it highlights inter-generational communication and understanding, promoting interactions among different age groups through shared activities and spaces. This enriches the lives of both senior and younger generations, fosters a sense of community awareness, and contributes to social harmony.

This cultural transformation plays a pivotal role in the development of a health care system for older adults within future communities. It prioritizes the physical and mental health of the older adults while aiming to enhance their overall well-being comprehensively. By equipping communities with diverse hardware and software resources, it aims to provide more comprehensive health care for older adults. By enhancing the digital literacy of the older adults, it helps them better integrate into the digital age, enjoying a more convenient and enriched life. Promoting inter-generational communication and community participation increases the older adults’ sense of belonging, thereby comprehensively improving their level of health care, mental health, and self-worth, creating more inclusive and interconnected communities. Ultimately, these cultural changes enable society to see the older adults as an indispensable part, providing comprehensive, efficient, and caring health care for older adults in future communities.

### 5.1 Challenges

However, it is noteworthy that this domain encounters challenges across various facets. Firstly, there lies the question of how health care for older adults within future communities can achieve sustainable development. Its industrial chain is elongated, characterized by significant investments and extended returns on investments. The construction fund is predominantly reliant on local financial support. This poses complexities in its advancement (28). Additionally, its construction and operation necessitate a digitized and systematic approach. This is a protracted process, and the importance of guiding, structuring, and coordinating sustainable and robust development of the project through long-term planning with multi-party participation cannot be overstated.

Secondly, a pressing concern is the education of specialized talent to ensure the effective integration of digital technology and services. The health care for older adults landscape within future communities necessitates a foundational support system built on skilled professionals. However, the current phase of development manifests a dire scarcity of resources in terms of digital technology professionals, integrated management experts, and specialized health care service providers. This talent shortage gravely jeopardizes the prospective development of health care for older adults within communities (29).

Thirdly, ensuring the older adults’ sense of digital inclusion and security is significant. A considerable portion of the elder population has limited cognition and comprehension of contemporary digital technologies and other current digital infrastructures. This often culminates in sentiments of distrust and resistance. How to effectively communicate information to the older adults and convey a sense of security in the digital age so that they can peacefully and actively participate in and enjoy the dividends brought by smart devices is also a key point in the future development of healthy aging in the community (30).

### 5.2 Call to action

**Figure 2:**
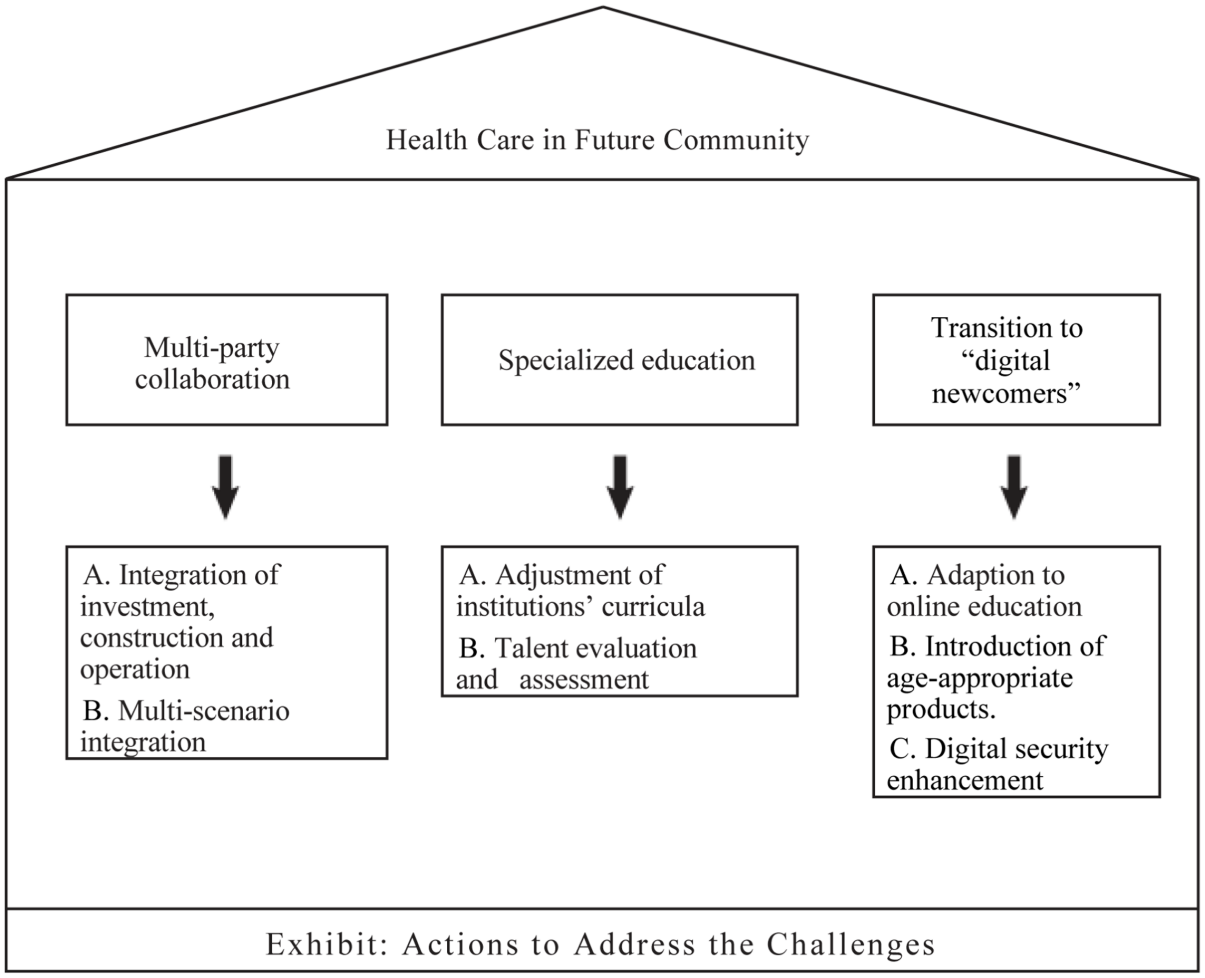
Actions to address the challenges.

To address those challenges, we propose the following recommendations (Figure 2): Firstly, it is crucial to actively deepen multi-party collaboration to ensure the sustainable development of community eldercare. On one hand, by integrating investment, construction, and operations, we can solidify the financial foundation. Leveraging a diversified investment framework, the unique expertise of both government and market forces should be fully utilized (31). Collaborative efforts should be emphasized, sharing the risks and revenues of future community eldercare investments (32). On the other hand, it’s crucial to integrate future community health care with scenarios such as transportation, entrepreneurship, and services, all of which offer clear development strategies and stable cash flows. The integration of smart modules can foster practical application across multiple scenarios, meeting different group needs while extracting both commercial and societal value. It is feasible to integrate the surrounding health care service resources with the local foundational health care facilities, the health care industry, and overall social development (33). For instance, a government-led training program for age-friendly infrastructure planning could be developed. This program would bring urban planners, architects, and community members together to participate in training focused on adapting community public spaces and buildings for the older adults. This collaborative approach would help create a safer, more convenient environment that benefits the older adults, planners, architects, and the entire community. By fully utilizing creative and industrial thought, an internal growth mechanism for future community health care for older adults care can be achieved. Establish a reward mechanism based on incremental value creation, ensuring that the economic value generated by future community health care projects is reflected in the returns of investment and financing projects, thereby achieving incentive compatibility and sustainable investment.

This solution is expected to have a positive impact on multiple fronts. Firstly, the introduction of smart modules and innovative solutions drives technological advancements in health care services, improving service efficiency, cost-effectiveness, and personalization to effectively address the public service needs of the community’s seniors. Secondly, through the integration of various services and multi-stakeholder collaboration, it creates a more tightly-knit community, enhancing the community’s cultural atmosphere and promoting social cohesion. This continuously improves the older adults’ sense of gain, happiness, and security, building a sense of belongings for seniors. Finally, the integration of the health care industry with other sectors contributes to the growth of the community industry, providing a sustainable source for community development. The investment return system ensures that economic value directly benefits the community, stimulating economic growth, creating more employment opportunities, and promoting entrepreneurial spirit. The successful implementation of the actions also provides experiences for other regions, enhancing the resilience of society in the face of aging challenges. Such a comprehensive and sustainable health care scheme benefits not only the elder population but also contributes to the overall well-being of the community, society, and economy.

Secondly, while focusing on educating specialized talents, we should establish a comprehensive system for evaluating and assessing talent. On one hand, higher education institutions should be encouraged to adjust their curricula in accordance with the future needs of community eldercare, producing professionals adept in areas like technological development, resource management, eldercare services, and specialized medical care. By introducing relevant courses, an effective fusion of academic education and vocational training should be realized, forming a sustainable talent cultivation mechanism (34). Furthermore, it is possible to establish training programs for healthcare professionals that focus on geriatric care and mental health. These programs can be developed in collaboration with local healthcare agencies, hospitals, and educational institutions. This initiative can enhance the quality of healthcare and mental health services provided to the older adults, offering vital support and specialized expertise for the development of future community health care for older adults. On the other hand, assessment, appraisal, and incentive mechanisms for specialized talent should be solidified, improving the compensation regulations and rights protection system for eldercare service providers (35). Multiple levels of professional technical positions should be established and specified, formulating policies for talent acquisition of I.T. professionals, management personnel, and eldercare service staff, thereby creating avenues for the growth of high-end talent. This will ensure the stability of future community health and eldercare construction.

This initiative yields several key positive outcomes. Firstly, it emphasizes the cultivation of professional talent, ensuring that community health care for older adults benefit from highly skilled professionals. This addresses the issue of underqualified personnel within the field, thereby enhancing service management efficiency. Communities will be equipped with professionals who possess expertise in both modern technology and health care, capable of addressing the diverse needs of the older adults, and providing personalized and efficient services. Secondly, it establishes an evaluation and incentive mechanism, addressing the shortage of personnel in community health care for older adults and ensuring the long-term stability of health care services. This not only aids in attracting talent to enter the health care industry but also motivates existing practitioners to continuously improve their professional competencies, thereby promoting an overall enhancement in the quality of health care services. This scheme not only benefits the older adults but also holds profound and positive implications for the entire community and social health care service system.

Lastly, efforts should be made to assist the older adults in bridging the “digital gap,” enhancing their sense of participation and security in the digital age. The goal is to transition them from being “digital refugees” to “digital newcomers.” Through institutions like senior universities or community education, the older adults should be encouraged to adapt to online learning (36). Project-based learning can enhance their digital integration, bolstering their digital skills and enthusiasm for community participation. For instance, hosting digital literacy workshops for seniors, a collaborative project involving community centers, libraries, and tech companies. This method can enhance the digital skills of senior citizens, enabling them to actively participate in the digital society and economy. Moreover, it also presents tech companies with opportunities to tap into the senior market and create commercial value for society. Family members can play a role in this education process, assisting the older adults in mastering digital tools, thereby mitigating the generational “digital gap.” Furthermore, it is essential to enhance digital friendliness for seniors by introducing age-appropriate products. When companies develop and refine intelligent health care products and programs, they should make them more intelligent, user-friendly, and comprehensive, in other words, reducing the learning barriers for the older adults. Moreover, digital security measures should be strengthened, respecting and ensuring the privacy rights of the older adults (37). Through community outreach and family support, awareness regarding digital fraud prevention among the older adults should also be raised, reinforcing their self-protection capabilities in the digital era. Financial institutions and community volunteer associations can collaborate to provide financial literacy training for seniors. Through this program, seniors can acquire greater financial knowledge, enhance their financial decision-making abilities, and better adapt to digital financial tools. The partnership between financial institutions and community volunteer associations will play a significant role in improving the economic well-being of the older adults.

The implementation of this initiative will bring various positive effects. Firstly, seniors will better integrate into the digital age, enabling them to use digital tools easier. They will have convenient access to online information, actively engage in digital social and entertainment activities, expand their social interactions, and thereby enhance their quality of life and sense of well-being. Secondly, the active involvement of family members will not only assist seniors in mastering digital technology but also promote communication and interaction among family members, strengthening family bonds and providing seniors with more familial warmth, thus reinforcing their sense of belonging within the family. Thirdly, digital security measures will equip seniors with greater digital security awareness, enabling them to better discern digital threats and prevent digital fraud, ensuring the safety and privacy of their digital lives. This initiative contributes to seniors’ adaptation to the digital age, active participation in the digital society, improved quality of life, and simultaneously fosters the digital development of society.

### 5.3 Limitation

Given that the concept of “future communities” is still nascent in China, it embodies an innovative endeavor that integrates various dimensions, including technological innovation, conceptual innovation, and service innovation. Due to its novel nature, obtaining information regarding these future communities becomes a challenging task. While the communities surveyed in the questionnaire may not strictly adhere to the definition of future communities, they already encompass some of its characteristics. Such communities have the potential to evolve in alignment with the concepts of future communities, as the developmental challenges and pathways they encounter resonate with the essence of what future communities represent.

## Data availability statement

The original contributions presented in the study are included in the article/supplementary material, further inquiries can be directed to the corresponding author.

## Ethics statement

Ethical review and approval was not required for the study on human participants in accordance with the local legislation and institutional requirements. Written informed consent from the patients/ participants or patients/participants’ legal guardian/next of kin was not required to participate in this study in accordance with the national legislation and the institutional requirements.

## Author contributions

BZ: Conceptualization, Formal analysis, Funding acquisition, Investigation, Methodology, Supervision, Writing – review & editing. QD: Conceptualization, Formal analysis, Investigation, Methodology, Writing – original draft. SZ: Writing – review & editing. DZ: Writing – review & editing.
